# Ceftazidime-induced urinary calculi in children: A case report

**DOI:** 10.1097/MD.0000000000042221

**Published:** 2025-04-18

**Authors:** Quan Liu, Zhike Huang, Kai Yi, Shigui Liu, Xiaoping Zhang, Maolin Xiao

**Affiliations:** a Department of Urology, Liuzhou Traditional Chinese Medical Hospital, Liuzhou, Guangxi Province, China.

**Keywords:** ceftazidime, ceftriaxone, children, drug-induced calculus, urinary calculus

## Abstract

**Rationale:**

Although drug-induced calculi are rare, numerous studies have reported on urinary tract stones caused by ceftriaxone, Urinary calculi induced by ceftazidime has only been reported in 1 study

**Patient concerns:**

The patient, an 8-year-old male, experienced pain in the lower right abdomen for 3 days and worsening fever for 1 day.The admission diagnosis was acute appendicitis.On the same day, the patient underwent laparoscopic appendectomy and was continuously given anti-infection treatment continuously by intravenous infusion of ceftazidime (1.0 g each time, every 8 hours, for 7 days). The patient’s condition improved, and he was discharged. One day later, the patient was readmitted to the hospital due to right lumbar pain. An abdominal computed tomography scan revealed that the patient had right lower ureteral stones, right kidney stones, and hydrops in the right pelvis and upper ureter.

**Diagnosis:**

Ceftazidime-associated urinary calculus.

**Interventions:**

In terms of the treatment plan, we did not continue to use ceftazidime for anti-infection treatment. The patient was given 500 mL of normal saline through intravenous injection, a phloroglucinol spasmolytic injection, and 1500 mL of water a day.

**Outcomes:**

Three days later, the patient excreted small gravel in the urine,and the physical and chemical properties of the stones were analyzed to provide a reference for clinical treatment.

**Lessons:**

We consider the possibility of drug-induced stones and the need to stop the drug in time after this diagnosis. After conservative treatment, patients can generally heal themselves and avoid unnecessary surgical intervention.

## 1. Introduction

Ceftazidime is a third-generation cephalosporin with broad spectrum activity against gram-negative bacilli, including *Pseudomonas aeruginosa* First introduced for clinical use in the 1980s, this antibiotic is now frequently used in patients.^[1]^ Side effects associated with ceftazidime use include liver injury and thrombocytopenia,^[2,3]^ with extremely rare cases of urinary stones. In 2020, Gao P First reported an 8-year-old boy who developed stones in both the left ureter and kidney after an intravenous infusion of ceftazidime. The calculi disappeared after intravenous ceftazidime was terminated, a double J-type ureteral stent was inserted surgically, potassium sodium hydrogen citrate granules were taken orally, and the urine was alkalized.^[4]^ In August 2023, a child with urinary calculi caused by the injection of ceftazidime was admitted to our hospital. After alkalization of the urine, spasmolytic treatment, drug discontinuation and hydration treatment, the stones were discharged.

## 2. Cases

**Figure 1.**
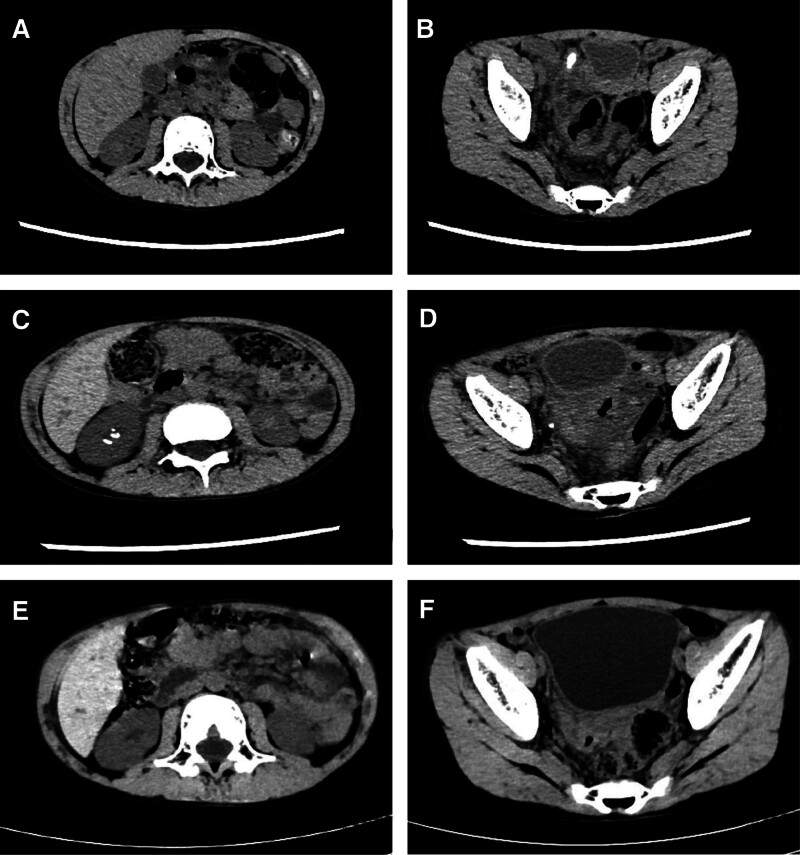
CT features during calculus treatment in the child: CT scan before treatment with ceftazidime showing no kidney stones (A) and no ureteral stones (B); CT scan 1 week after treatment with ceftazidme showing kidney stones (C) and ureteral stones (D); no kidney stones (E) or ureteral calculi (F) were detected by CT after treatment. CT = computed tomography.

The patient, an 8-year-old male, was admitted to our hospital on August 9, 2023, due to pain in the lower right abdomen for 3 days and worsening fever for 1 day. On August 9, 2023, an ultrasound scan of the child indicated the possibility of acute appendicitis, and a lower abdominal computed tomography (CT) image showed (Fig. 1A, B): revealed acute suppurative appendicitis and highly suspected perforation, combined with local peritonitis and appendicitis. A routine blood test revealed that the leukocyte count was 22.9×10^9^/L. The admission diagnosis was acute appendicitis, and the patient had no other medical conditions. On the same day, the patient underwent laparoscopic appendectomy and was continuously given anti-infection treatment by intravenous infusion of ceftazidime (1.0 g each time, every 8 hours, for 7 days). The patient ate normally, and the white blood cell count was normal the next day after surgery. The patient did not develop sepsis during treatment. The patient’s electrolytes (K^+^, Na^+^, Cl^−^, Ca^2+^, Mg^2+^, and P) and albumin levels were all normal during the hospitalization, and there was no metabolic disorder or malnutrition. The patient’s condition improved, and he was discharged.

**Figure 2.**
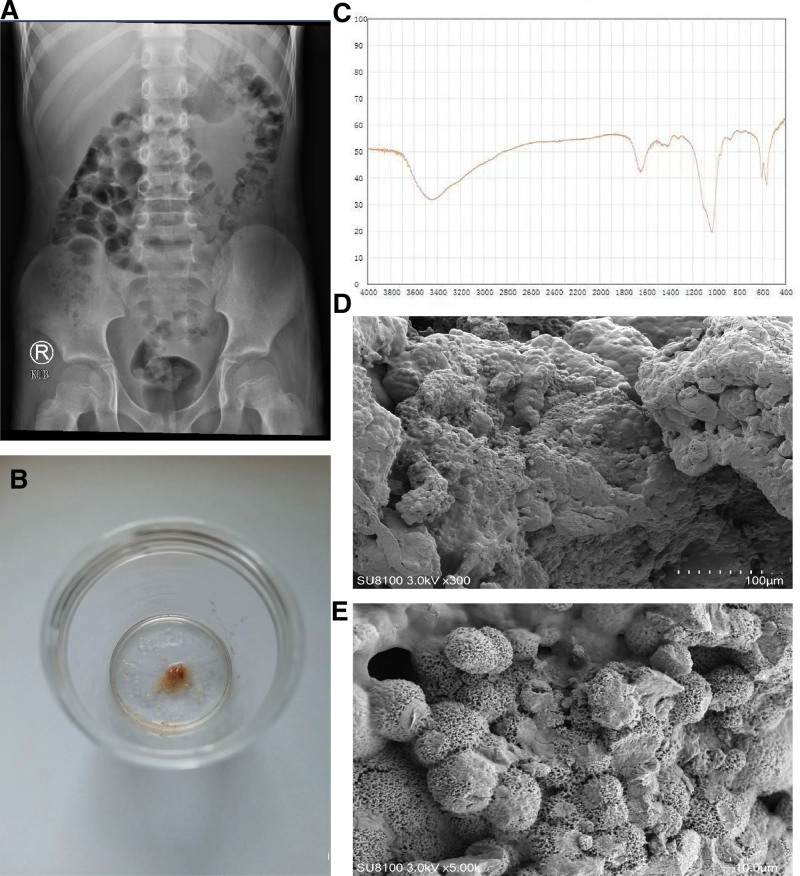
Physical and chemical characteristics of the stones: urinary system plain film, no stone development (A); the stones discharged by the child are small and gravel-like sand (B); composition analysis of the stones, and the main components are carbonate apatite (90%) and calcium oxalate monohydrate (10%) (C); observation under scanning electron microscopy showed a fragile, rough surface of stones (×300) (D); and calculi with many prominent hemispherical structures with a loose and fragile surface (×5000) (E).

On August 17, 2023, the patient was readmitted to the hospital due to right lumbar pain. An abdominal CT scan revealed that the patient had right lower ureteral stones, right kidney stones, and hydrops in the right pelvis and upper ureter (Fig. 1C, D). The average CT value of the right lower ureter calculi was 263.75 ± 46.56 HU, and the size of the stone was 4 mm × 4 mm × 10 mm. The average CT value of the right renal calculi was 231.00 ± 49.29 HU), and the size of the stone was 6 mm × 5 mm × 7 mm. The plain film of the patient^,^s urinary system did not reveal any stones (Fig. 2A). In terms of the treatment plan, we did not continue to use ceftazidime for anti-infection treatment. The patient was given 500 mL of normal saline through intravenous injection, phloroglucinol spasmolytic injection, and 1500 mL of water a day. During hospitalization, the patient retained for 24 hours with a urinary calcium of 3.19 mmol, a blood calcium level of 2.46 mmol/L, and a blood creatinine level of 41.2 µmol/L, which are all within the normal range. The patient excreted small gravel in the urine 3 days later (Fig. 2B). A component analysis of some stones (Fig. 2C) revealed the presence of carbonate apatite (90%) and calcium oxalate monohydrate (10%). part of which was taken for scanning electron microscopy (Fig. 2D, E). We took 0.5 g of the same batch of ceftazidime (China, Zhejiang Jutai Pharmaceutical, T172305451) used by the patient and mixed it with deionized water (China, Shanghai, Suitiankeji, conductivity of 0.055 µs/cm or less, resistivity = 18.25 MΩ*cm, pH ≤ 7). The mixture was diluted to 100 mg/mL. Ceftazidime stock solutions of with different concentrations were added to the solutions containing 5 mM calcium chloride (China, Shanghai, HUSHI, 20230414). The final concentrations were 0.5, 1.0, 2.0, 4.0, 8.0, and 16.0 mg/mL, respectively; and the reaction mixtures were incubated at 25°C for 2, 6, 12, 24, 36, 48, and 72 hours. The solutions were observed under an inverted microscope, but no crystals formed. On August 23, 2023, a urinary CT examination revealed that the stones in the right kidney and ureter had disappeared (Fig. 1E, F), and the patient was cured and discharged. The patient was followed up until August 31, 2024, and had no symptoms, such as low back pain or abdominal pain. A CT scan of the urinary system revealed no recurrence of calculi.

## 3. Discussion

Drug-induced urinary calculi are rare.^[5]^ Previous studies reported that ceftriaxone, a ceftriaxone drug, can cause urinary calculi. The mechanism may involve the binding of ceftriaxone to calcium ions, leading to the formation of crystals that closely adhere to the surface of renal tubule cells, thereby blocking renal tubules, damaging cells, and interfering with calcium reabsorption. The increased calcium level in the urine continues to bind to ceftriaxone and form more crystals.^[6]^ Although ceftazidime causes urinary calculi, only 1 study reported that calculi disappeared after the insertion of a double J-type ureteral stent during surgery and the cessation of intravenous injection of ceftazidime and potassium sodium hydrogen citrate particles to alkalize the urine.^[4]^ The patient underwent laparoscopic appendectomy due to acute appendicitis. Preoperative CT revealed no urinary calculi, and the patient only received only anti-infection treatment with ceftazidime without any other drug injection or drug administration, The patient had no metabolic disorders, malnutrition or other diseases. Therefore, we believe that the calculi in the right kidney and ureter of the patient were caused by ceftazidime. In addition, we refer to the treatment plan of ceftriaxone causing urinary calculi^[6]^ and recommend ceftazidime drug discontinuation, spasmolytic treatment, and hydration treatment before calculus discharge.

Most of drug-induced stones are transparent and not visible on plain abdominal films but can be detected by ultrasound or CT.^[7]^ In this case, the plain abdominal film of the patient did not show stone shadows, while as CT could clearly revealed stones with columnar changes in the ureter. The CT value of the stones was low and close to that of ceftriaxone stones.^[8]^ After the stone was discharged, analysis revealed that it was small in size, sandy, had a loose texture, and a partly granular appearance, which is consistent with the characteristics of ceftriaxone stones.^[7]^ Composition analysis of stones is an important prerequisite for studying their pathogenesis. The results revealed that the main component was carbonate apatite. We referred to the in vitro synthesis of ceftriaxone stones,^[9]^ but crystals were not formed, so comparisons with ceftazidime stone samples could not be made.

A high urine calcium content can promote the formation of ceftriaxone stones in the body. Ceftriaxone sodium does not undergo biological transformation in the body and is excreted as a prototype through the kidneys (approximately 2/3), and the biliary tract (1/3). As such, ceftriaxone sodium is present at high concentrations in the bile and kidneys and can form insoluble ceftriaxone calcium precipitates after it combines with calcium ions. In particular, large doses of long-term use can quickly lead to stones formation in the bile duct, gallbladder, and kidney collection system.^[10]^ However, whether ceftazidime stone formation is related to calcium ions in the body remains unclear. The blood calcium and 24-hour urinary calcium levels of the patients were normal, so we could not conclude that the presence of ceftazidime stones was related to the concentration of calcium ions in the body, which may be related to the small number of cases. The scanning electron microscopy of ceftriaxone stones revealed a fragile and rough surface with many prominent hemispherical structures, and the surface of the hemispherical structures was loose and fragile.^[8]^ A small portion of the stones this study’s patient was taken for scanning electron microscopy, and their characteristics were similar to those of ceftriaxone stones.

At present, the mechanism by which ceftazidime causes the formation of urinary stones remains unclear. When we use ceftazidime in clinical practice, especially in children, we should avoid high concentrations, large doses, and long-term use and should monitor the occurrence of waist distension, kidney colic, abdominal pain, nausea, vomiting, and other related symptoms during treatment. In these cases, we should consider the possibility of drug-induced stones and stop the drug in time after diagnosis. After conservative treatment, patients can generally heal themselves and avoid unnecessary surgical intervention. When bilateral ureteral obstruction is severe, bilateral hydronephrosis affects kidney function. The obstruction can be removed through surgery, such as ureteral intubation and laser lithotripsy.

## Acknowledgments

We are especially grateful to the patient’s mother for agreeing to have the stones passed for further analysis.

## Author contributions

**Conceptualization:** Quan Liu.

**Data curation:** Quan Liu, Zhike Huang, Kai Yi.

**Formal analysis:** Quan Liu, Zhike Huang, Kai Yi, Shigui Liu.

**Funding acquisition:** Quan Liu, Xiaoping Zhang.

**Software:** Quan Liu, Xiaoping Zhang.

**Writing – original draft:** Quan Liu.

**Writing – review & editing:** Quan Liu, Xiaoping Zhang, Maolin Xiao.
